# Linking Tree and Shrub Biomass to Plant Diversity During Early‐Stage Alpine Afforestation

**DOI:** 10.1002/ece3.71842

**Published:** 2025-08-27

**Authors:** Chun‐Jing Wang, Wu‐Xian Yan, Shan‐Feng Huang, Dong‐Zhou Deng

**Affiliations:** ^1^ Ecological Conservation, Restoration and Resource Utilization on Forest and Wetland Key Laboratory of Sichuan Province, Sichuan Academy of Forestry Chengdu China; ^2^ Sichuan Giant Panda National Park Observation and Research Station; Wolong Forest Ecology Observation and Research Station of Sichuan Province Sichuan Aba China; ^3^ Climate Bridge Ltd Shanghai China

**Keywords:** aboveground biomass, afforestation, alpine ecosystem, general linear mixed model, phylogenetic diversity, species diversity

## Abstract

Afforestation has considerable potential to restore and maintain plant diversity, which is closely associated with ecosystem functions and services. However, there remain numerous uncertainties regarding alpine afforestation performance. Hence, it is necessary to determine the factors contributing to plant diversity during the early stages of afforestation in alpine regions. Our main objective of this study was to examine the effects of tree and shrub biomass on plant diversity, namely, species (SD) and phylogenetic (PD) diversity, during the early period of afforestation. We used a general linear mixed model (GLMM) to determine the associations between tree and shrub biomass and multiple indices (Shannon, Simpson's diversity, species richness, Pielou's evenness, Faith's phylogenetic diversity, and net relatedness) at two spatial scales (1 × 1 m^2^ and 10 × 10 m^2^), based on 78 10 × 10 m^2^ sites, each containing five 1 × 1 m^2^ plots. On the basis of the GLMM results, we established that both tree and shrub biomass had significant effects on plant diversity at the site and plot scales, and found that the responses of SD and PD to tree and shrub biomass were non‐linear. Overall, whereas at the site scale, SD (i.e., Pielou's evenness index) was highest at a median level of tree biomass, there were negative relationships between shrub biomass and the Shannon index, species richness, and Faith's PD at the plot scale. The effects of biomass on SD and PD were found to be dependent on different spatial scales (i.e., plot and site) and life form (i.e., tree or shrub), thereby providing further evidence regarding the efficacy of ecological restoration subsequent to alpine afforestation. The findings of this study will provide a novel empirical reference for optimal tree species selection and planning for planting practices in alpine regions.

## Graphical abstract

The main objective of our study was to explore the effects of tree and shrub biomass on plant diversity, namely, species diversity (SD) and phylogenetic diversity (PD), under early‐stage afforestation. The effects of biomass on SD and PD were found to be dependent on different spatial scales (i.e., plot and site) and life form (i.e., tree or shrub), thereby providing further evidence regarding the efficacy of ecological restoration subsequent to alpine afforestation.

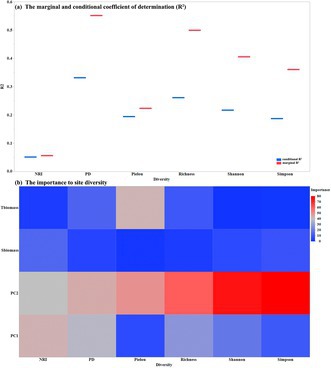

## 1 Introduction

By establishing new forests on previously unforested land (e.g., abandoned agricultural land), afforestation represents an effective approach for retaining plant diversity and maintaining valuable ecosystem functions and services (Andrés and Ojeda 2002; Fiandino et al. 2018; Acharya et al. 2019). A range of different control measures, mainly artificial afforestation, has been implemented with a view toward the restoration of degraded alpine lands, and numerous studies have assessed the effects of afforestation on ecosystem functions and services (e.g., soil sequestration; Zaloumis and Bond 2011; Acharya et al. 2019; Segura et al. 2021). Assessing the effects of the early stages of afforestation on plant diversity can contribute to forest development and sustainability in alpine regions (Baltzinger et al. 2011; Souza et al. 2013; Chen et al. 2020; Segura et al. 2021). Following plantation establishment, plant communities are progressively replaced by species characteristic of shady environments and disturbed soils, particularly rushes and grasses (Björk and Molau 2007; McKee et al. 2007; Zaloumis and Bond 2011; Segura et al. 2021). Alpine ecosystems are notably sensitive to environmental change (Ernakovich et al. 2014), and plantations in alpine regions are particularly susceptible to disturbances (Bebi et al. 2017; Baughman et al. 2020). Consequently, there are uncertainties regarding the performance of early‐stage alpine afforestation, an effective assessment of which can guide policymakers in making economic and equipment investment decisions for plantation forests (Farley et al. 2005; Souza et al. 2013). However, the extent to which changes in plant diversity occur during land management during the early stages of afforestation has yet to be sufficiently established.

Given the effects of planted tree density on species interactions and composition, the dimensions (diameters and heights) of tree and shrub species can potentially have a significant influence on understory plant diversity (Pukkala et al. 2013; Forrester and Tang 2016; del Río et al. 2019). Moreover, in addition to the density of trees and shrubs within plantations, the plant community structure may differ with diameter and height (Forrester and Tang 2016; Zambrano et al. 2019). Consequently, the appropriate selection of tree and shrub species according to their diameter and height characteristics is important for the development and implementation of environmentally sound alpine afforestation strategies (Feng et al. 2022). Restoration and conservation performance may be influenced to differing extents by different tree species following alpine afforestation (Pommerening and Murphy 2004; Forrester and Tang 2016), and in this regard, biomass models have been widely developed to quantify the aboveground biomass of woody plants (i.e., trees and shrubs) based on their diameter and height (Luo et al. 2020; Wang et al. 2021). Accordingly, the biomass of trees and shrubs (as determined from plant diameter and height, and plantation density) can serve as an integral indicator for the assessment of plant diversity during the early stages of afforestation. However, few studies have assessed the effects of tree and shrub biomass on plant diversity during this phase of forest development.

Furthermore, given that numerous authors (e.g., Morlon et al. 2011; Pio et al. 2011; Winter et al. 2013; Rodrigues and Gaston 2002) have attempted to incorporate phylogenetic diversity into conservation planning, we sought in the present study to examine the effects of tree and shrub biomass on plant diversity, namely, species (SD) and phylogenetic (PD) diversity, during the early stages of afforestation. We focused specifically on alpine regions, the ecosystem of which is particularly vulnerable to environmental changes (e.g., deforestation, soil erosion, and climate change) (Björk and Molau 2007; Cui and Graf 2009; Ernakovich et al. 2014; Wan et al. 2023). Previous prediction studies have shown that alpine ecosystem functions and services may be lost owing to declining plant diversity (Cardinale et al. 2002; Gamfeldt et al. 2013; Dee et al. 2019). Furthermore, alpine deforestation can promote the redistribution of species, which may have a detrimental influence on biodiversity (Guo et al. 2018; Garcés‐Pastor et al. 2022). The negative effects of rapid environmental change on biodiversity may be enhanced in alpine landscapes owing to the sensitivity of species distribution to ecological variation (Björk and Molau 2007; Cui and Graf 2009; Guo et al. 2018; Garcés‐Pastor et al. 2022). Hence, an assessment of the effects of afforestation on plant diversity can contribute to enhancing the efficacy of ecological restoration and recovery in alpine regions.

In this study, we sought to verify the following two hypotheses. During the early stage of afforestation (1) tree and shrub biomass would have significant effects on SD and PD and (2) such effects would differ between trees and shrubs. To test these hypotheses, we used linear mixed models to examine the relationships between tree and shrub biomass using multiple indices of diversity, namely, the Shannon index, Simpson's diversity index, species richness, Pielou's evenness index, Faith's PD, and the net relatedness index (NRI) at two spatial scales (1 × 1 m^2^ and 10 × 10 m^2^) based on 78 (10 × 10 m^2^) sites, each encompassing five (1 × 1 m^2^) plots.

## 2 Materials and Methods

### 2.1 Study Region

**FIGURE 1:**
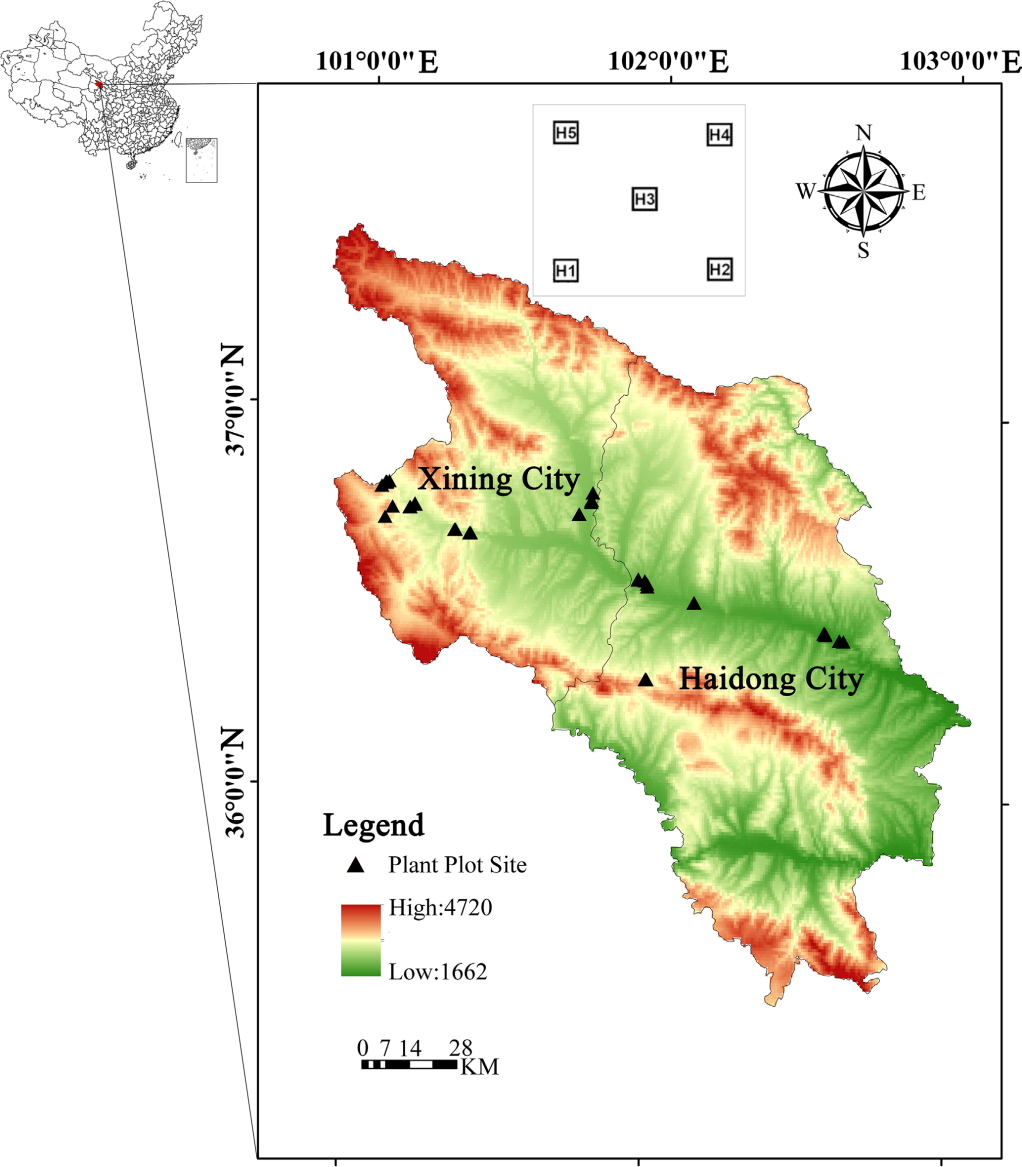
A map of the study sites within the Huangshuihe River Valley in Qinghai Province, China. In each study area, we investigated six sites. In each of these sites, with due north as a reference, plots 1, 2, 3, and 4 are set from right to left in four counterclockwise corners, and plot 5 is sited in the center of the investigation site. The color from greed to red indicates the increasing altitude (m) of study sites.

The study region, which ranges in elevations of between 1700 and 3800 m, is located in Qinghai Province within the northeastern part of the Qinghai–Tibet Plateau, China, and includes the Yellow River–Huangshui River Valley (Figure 1) (Zhao et al. 2013; Liu et al. 2021). It is not only an important part of the Qinghai–Tibet Plateau but also the most representative region in terms of biodiversity (Feng et al. 2021). The mainstem has a total length of 374 km with a drainage area of 17,733 km^2^ (Wang et al. 2022). The study region covers most of the upper reaches of the Huangshui River Basin in Minhe County, with a mainstem length of 278 km, spanning an area demarcated by the coordinates 36°02′N–37°28′N and 100°42′E–103°01′E, and is characterized by a plateau arid and semi‐arid continental climate (Liu et al. 2021; Wang et al. 2022). The annual average precipitation is 381.1 mm, and the annual average temperature ranges from 3.1°C to 7.9°C (Feng et al. 2021). The main conservation and restoration activities in the Huangshui River Basin are afforestation for the provision of ecosystem functions and services (Zhao et al. 2013; Feng et al. 2021; Liu et al. 2021).

Fieldwork was undertaken as early as 10–15 years after the date of planting, corresponding to the early‐stage period of afforestation (Figure 1). During the third phase of the Xining Nanbei Mountain Greening Project (2005–2013), the area of artificial plantation reached 5668.22 ha, the majority of which is located in the study area, in which forest vegetation cover has rapidly developed on hitherto barren hillsides in the Huangshui River Basin. The implementation of greening projects has, to a certain extent, enhanced the comprehensive environmental quality of the Huangshui River Basin and the ecosystem service capacity of green barriers (Li et al. 2015).

### 2.2 Sampling Design

For the purposes of this study, we investigated 13 study areas, each of which was defined by a circle of 10‐km radius (Fang et al. 2009; Figure 1), and was characterized by similar environmental conditions (e.g., soil and climate), and were collectively distributed over the entire elevational range of the Huangshui River Valley (Feng et al. 2021). As representative environmental characteristics, we used climate, elevation, and soil. Climate data, comprising four bioclimatic variables [annual mean temperature (Bio1), temperature seasonality (Bio4), annual precipitation (Bio12), and precipitation seasonality (Bio15)] were downloaded from the WorldClim database (https://chelsa‐climate.org/). Data for five soil variables, namely, soil organic carbon content, pH in H_2_O and KCl solutions, and the contents of sand, silt, and nitrogen, were downloaded from the SoilGrids database (www.soilgrids.org; Hengl et al. 2017). The resolution of all environmental data was 2.5 arcminutes (~4.3 km at the square). Using these variables, principal component analysis (PCA) was used to quantify the environmental conditions based on the study sites and plots, and we used the first (PC1) and second (PC2) principal component scores to assess the environmental space for each site. Specifically, PC1 and PC2 were found to explain 70.008% of the total variation in the environmental factors for the sites. PC1 explained the variation in soil organic carbon, nitrogen, sand, and silt contents, and PC2 explained the increasing annual mean temperature and temperature seasonality and declining precipitation seasonality.

In each study area, we investigated six sites, in which we selected one to three typical natural or semi‐natural target habitat types (e.g., dry forest and shrubland, temperate coniferous forest, deciduous forest, and semi‐natural grassland). The selection of habitat types was based on their relatively high frequency of occurrence in the study area (i.e., very rare and extreme habitats were avoided). Within each study area, one to three typical target habitats were sampled as pairs of specific sites (10 × 10 m^2^), each of which had been subjected to different anthropogenic disturbance (relatively natural vs. disturbed). For each 10 × 10 m^2^ site, we established five 1 × 1 m^2^ plots (Figure 1). Each site had a nested structure, such that sampling was conducted at two scales, within a core plot (i.e., the midpoint) and within four surrounding plots, following the methods described by Fang et al. (2009) (Figure 1).

We assessed the effects of afforestation at two spatial scales (1 × 1 m^2^ and 10 × 10 m^2^) based on the different spatial structures and compositions of the plantation forests. In most cases, we assessed SD and PD within the floor and understory layers on a 1 × 1 m^2^ plot scale, and the whole SD and PD on a 10 × 10 m^2^ plot scale, thereby enabling us to examine the regulation of plant diversity by tree and shrub biomass during the early phase of alpine afforestation at different spatial scales.

### 2.3 Estimation of Plant Diversity and Biomass

We estimated plant diversity based on SD and PD at two spatial scales (1 × 1 m^2^ and 10 × 10 m^2^), using the Shannon index, Simpson's diversity index, species richness, and Pielou's evenness index to quantify the SD of plants, whereas PD was used as a measure of biodiversity that incorporates both the number of species and their evolutionary histories based on a phylogenetic tree of vascular plants according to three different botanical nomenclature systems. Values for the Shannon, Simpson, species richness, and Pielou indices were obtained using equations described by Fang et al. (2009). Faith's PD is a commonly used measure of the phylogenetic diversity of species assemblages and was implemented using the V.PhyloMaker2 package in the R statistical computing environment. NRI is the most commonly used measure of phylogenetic relatedness, for which a positive value is taken to be indicative of the phylogenetic clustering of species, whereas a negative NRI value indicates phylogenetic overdispersion. Faith's PD and NRI were quantified as described by Jin and Qian (2022).

To estimate the biomass of trees and shrubs, we measured aboveground biomass based on the biomass equations described by Luo et al. (2020) and Wang et al. (2021). With respect to tree biomass, the predictor variables for the biomass equations were limited to tree diameter at certain heights (i.e., basal diameter and diameter at breast height 1.3 m above the soil surface), tree height, and a combination of both. With respect to shrub biomass, height, basal diameter, and crown projection were recorded during fieldwork. The equations used to infer tree and shrub biomass have been described by Luo et al. (2020; Table S1) and Wang et al. (2021; Table S2). All the data on SD, PD, and biomass were log transformed prior to our analysis.

### 2.4 Data Analysis

We used intra‐class correlation coefficients to validate model structure and differences in biomass and diversity at the site scales based on the 13 study areas, and used a general linear mixed model (GLMM) to examine the relationships between tree and shrub biomass and SD (Shannon, Simpson, species richness, and Pielou) and PD (Faith's PD and NRI). The marginal and conditional coefficients of determination (*R* ^2^) of the GLMM were used to determine the potential effects of tree and shrub biomass on SD and PD, for which the tree and shrub biomass of the study sites and PCs 1 and 2 were used as fixed factors, and study areas served as random factors. Thus, we excluded the random effects of the study area from our analysis. Both marginal and conditional *R* ^2^ values were high, indicating strong associations between tree and shrub biomass at the study sites and PCs 1 and 2 with SD and PD. We determined the individual contribution of tree and shrub biomass of the study sites and PCs 1 and 2 to site diversity, namely, SD and PD, based on the methods described by Lai et al. (2022a, 2022b).

To quantify the plot diversity of each site based on data from the six study plots, we averaged the values of SD and PD, and linear, binary, and triple regression models were used to quantify the changing trends in SD and PD across a gradient of tree and shrub biomass based on site and plot diversity, respectively, depending on the highest values of *R* ^2^. The results of the regression models with *R* ^2^ values higher than 0.1 and *p*‐values lower than 0.1 were considered for analysis. We compared the contributions of tree and shrub biomass to SD and PD based on site and plot diversity.

All the analyses were conducted using the packages glmm.hp, lme4, and vegan in the R statistical computing environment (https://www.r‐project.org/).

## 3 Results

**FIGURE 2:**
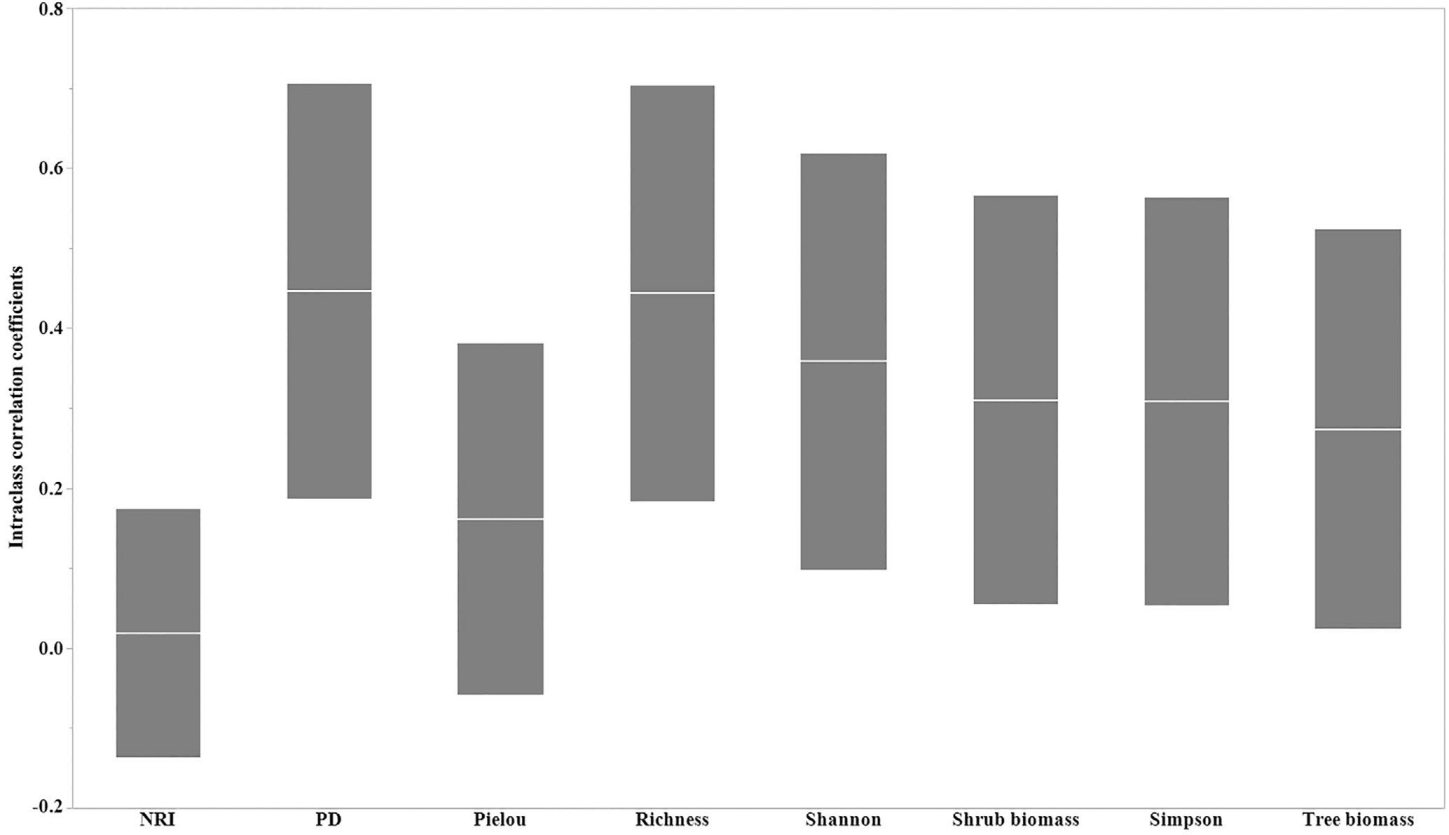
Intra‐class correlation coefficients of tree and shrub biomass, species diversity (SD), and phylogenetic diversity (PD) based on the 13 study sites. Boxes show the intra‐class correlation coefficients (white lines), and lowest and upper (borders).

**FIGURE 3:**
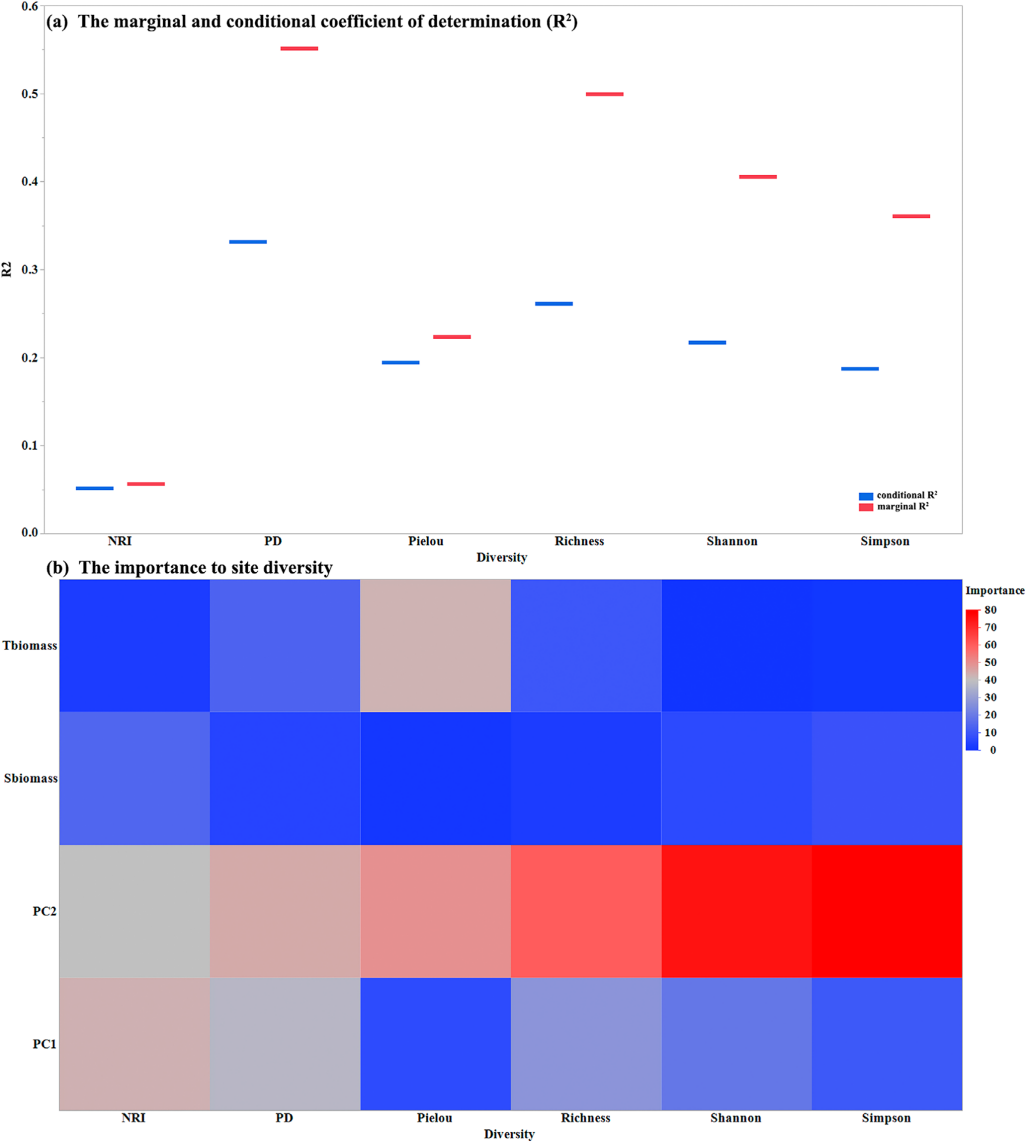
The marginal and conditional coefficient of determination (*R* ^2^) of general linear mixed models of plant diversity, including species diversity (SD) (assessed using the Shannon index, Simpson's index, species richness, and Pielou's evenness) and phylogenetic diversity (PD) (assessed using Faith's PD and the net relatedness index [NRI]) with tree and shrub biomass and environmental conditions (PCs 1 and 2) at the site scale (a); The importance of tree and shrub biomass and environmental conditions to SD and PD (b). The blue lines represent conditional *R* ^2^ and the red lines represent conditional *R* ^2^ in panel (a). The color from blue to red represents increasing importance of tree and shrub biomass and environmental conditions (PCs 1 and 2) to SD and PD. PC1 explains the soil factors including soil organic carbon, nitrogen, sand, and silt contents, and PC2 explains the climatic factors including annual mean temperature and temperature seasonality and declining precipitation seasonality. Tbiomass and Sbiomass represent tree and shrub biomass respectively.

We found differences in plant biomass and diversity at the site scale among the different study areas (intraclass correlation coefficients < 0.5), with the strongest intraclass correlations detected for PD and species richness (Figure 2). On the basis of the marginal and conditional *R* ^2^ values, both tree and shrub biomass, coupled with environmental factors, were established to have significant associations with SD (Shannon, Simpson, species richness, and Pielou) and PD (Faith's PD and NRI) at the site and plot scales (Figure 3). Environmental conditions (i.e., climate and soil) had larger effects on Simpson, Shannon, and species richness, whereastree and shrub biomass had relatively smaller effects at the site scale (Figure 3). Although climatic and soil factors had strong effects on SD and PD, tree biomass was established to make an important contribution to Pielou's index and Faith's PD, and shrub biomass was important for NRI at the site scale (Figure 3).

**FIGURE 4:**
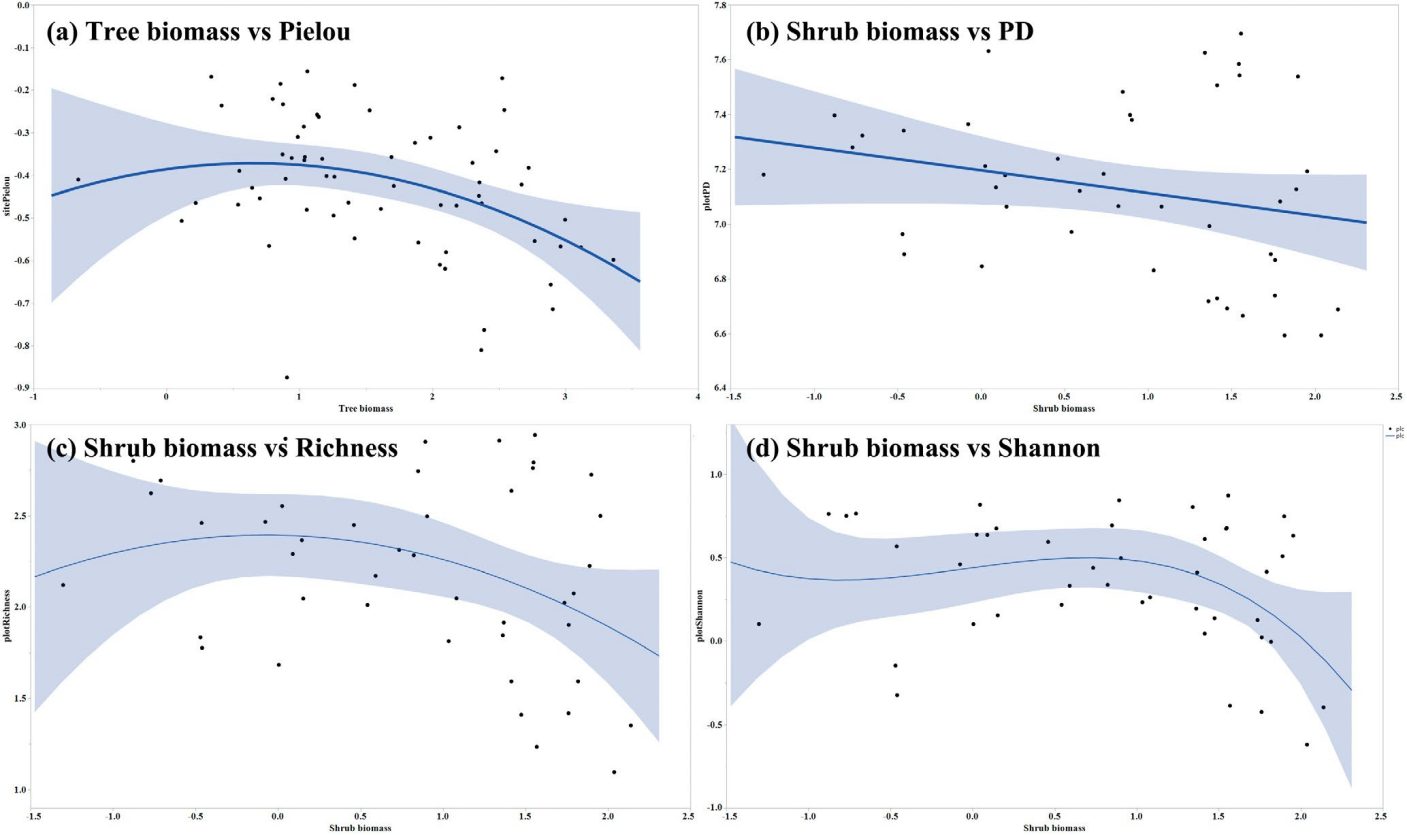
Associations between the biomasses of trees and shrubs biomass and species (SD) and phylogenetic (PD) diversities at the site and plot scales. Shaded areas over the dashed regression lines represent the 95% confidence interval of fitted index values. Site represents SD at the site scale and plot represents SD and PD at plot scale. (a) Association between tree biomass and Pielou's evenness, (b) Association between shrub biomass and PD, (c) Association between shrub biomass and species richness, and (d) Association between shrub biomass and Shannon index.

In addition, at the site scale, we found that tree biomass had strong effects on Pielou's index, whereas shrub biomass had strong effects on the Shannon index and species richness at the plot scale (Table 1). However, although we identified a significant relationship between shrub biomass and PD at the plot scale, this association was found to be relatively weak (Table 1). At both site and plot scales, the responses of SD and PD to tree and shrub biomass were non‐linear (Figure 4; Table 1). These findings revealed that at the site scale, there were initial increases and subsequent declines in the values of Pielou's index with increasing tree biomass (Figure 4). Overall, we established that at the plot scale, there were negative associations between shrub biomass and diversity assessed using the Shannon index and species richness (Figure 4).

**TABLE 1:** The associations between tree and shrub biomass and plant diversity including species diversity (SD) (as assessed using the Shannon index, Simpson's index, species richness, and Pielou's evenness) and phylogenetic diversity (PD) (as assessed using Faith's PD and the net relatedness index (NRI)) based on linear, binary, and triple regression models Bold values represent the linear, binary, and triple regression models with *R* ^2^ > 0.1 and *p* < 0.1.

	Linear				Binary				Triple			
	Tree		Shrub		Tree		Shrub		Tree		Shrub	
	*R* ^2^	*p*	*R* ^2^	*p*	*R* ^2^	*p*	*R* ^2^	*p*	*R* ^2^	*p*	*R* ^2^	*p*
*Site scale*												
Shannon	0.04	0.11	0.01	0.49	0.07	0.11	0.08	0.18	0.07	0.22	0.08	0.33
Simpson	0.05	0.07	0.00	0.85	0.09	0.05	0.09	0.16	0.10	0.11	0.09	0.30
Richness	0.00	0.70	0.03	0.28	0.01	0.70	0.04	0.39	0.01	0.87	0.05	0.58
Pielou	**0.12**	**0.01**	0.00	0.85	**0.15**	**0.01**	0.10	0.12	**0.15**	**0.02**	0.10	0.24
PD	0.01	0.39	0.02	0.36	0.03	0.39	0.02	0.63	0.04	0.54	0.03	0.79
NRI	0.00	0.62	0.01	0.60	0.01	0.73	0.01	0.86	0.03	0.55	0.01	0.94
*Plot scale*												
Shannon	0.02	0.26	0.05	0.14	0.02	0.51	0.12	0.07	0.03	0.68	**0.14**	**0.10**
Simpson	0.02	0.29	0.02	0.33	0.02	0.55	0.11	0.10	0.02	0.72	0.14	0.11
Richness	0.02	0.23	0.08	0.06	0.02	0.48	**0.12**	**0.08**	0.03	0.67	0.14	0.12
Pielou	0.02	0.24	0.00	0.92	0.03	0.45	0.08	0.20	0.03	0.63	0.09	0.30
PD	0.01	0.38	**0.06**	**0.10**	0.01	0.65	0.08	0.17	0.02	0.73	0.10	0.22
NRI	0.00	0.95	0.00	0.96	0.01	0.75	0.05	0.35	0.02	0.71	0.05	0.53

## 4 Discussion

Our findings in this study revealed significant associations between the biomass of trees and shrubs and species and phylogenetic diversities at both site and plot scales, thereby providing evidence to indicate that tree and shrub biomass may make a positive contribution to determining plant diversity during the early stages of afforestation. It has previously been established that assessments of ecosystem restoration and conservation can provide valuable information for enhancing the efficacy of environmental management during the initial phase of afforestation (Zaloumis and Bond 2011; Mori et al. 2017; Liu et al. 2021), and with relevant data and analyses, researchers can select appropriate plans for ecosystem restoration and conservation based on the regulation of plant diversity (i.e., SD and PD) by tree and shrub biomass during this period of forest development. Given such information, policymakers can integrate SD coupled with evolutionary processes into ecosystem restoration and maintenance decisions. Environmental conditions (i.e., climate and soil) were found to have significant effects on species diversity, as assessed using the Shannon, Simpson, and species richness indices. We established that tree and shrub biomass made substantial contributions to SD and PD. Specifically, we obtained evidence to indicate that high precipitation levels can enhance the association between tree biomass and species diversity. Our findings accordingly provide a valuable reference for the selection of tree species and planting approaches based on tree and shrub biomass.

At the site scale, we found that tree biomass influences SD, as indicated by an initial increase and subsequent decline in Pielou's index values in response to an increase in tree biomass. In this context, the findings of previous studies have indicated that tree height and diameter should be considered in afforestation planning (e.g., Schönenberger 2001; Gardiner et al. 2004; Cogliastro et al. 2003; Pukkala et al. 2013). Furthermore, planting density has been established to have notable effects on tree growth during the early stages of afforestation (Forrester and Tang 2016; Fiandino et al. 2018). Consequently, tree growth models should be developed to assess plantation performance (Hall et al. 2011; Jactel et al. 2017). With the development of biomass equations and height–diameter models for trees and shrubs (Luo et al. 2020; Wang et al. 2021), it is necessary to assess the regulation of SD by tree biomass during the early stages of afforestation. On the basis of our findings in this study, we recommend that to promote plant diversity, tree biomass should be maintained at a suitable density level through selective thinning, which involves the removal of branches or entire trees to enhance the health and growth of the remaining trees, often in forest or woodland settings (Duguid and Ashton 2013; Gamfeldt et al. 2013; Wan and Wang 2023). This practice reduces competition for resources, such as sunlight, water, and nutrients, thereby promoting stronger and more robust growth. Furthermore, promoting a high plant diversity has considerable potential for the restoration and maintenance of ecosystem functions and services (e.g., food, water, timber, air purification, soil formation, and pollination; Gamfeldt et al. 2013; Mori et al. 2017; Dee et al. 2019). Accordingly, given that a high biomass of trees is an important indicator of ecosystem function (Gamfeldt et al. 2013; Van Der Plas 2019), we suggest that the control of tree biomass should be considered in afforestation management.

Contrastingly, we found that shrub biomass contributes to SD and PD at the plot scale, although the observed effects were less pronounced than those associated with tree biomass and differed from the effects on site diversity. Notably, however, our findings tend to indicate that shrub biomass is negatively associated with plant diversity. Accordingly, shrub encroachment should be controlled during the early stages of afforestation. Thus, although individual shrub patches can have positive effects on multiple ecosystem services, ecological restoration may be negatively affected by shrub encroachment (Wilcox et al. 2018; Acharya et al. 2019; Geissler et al. 2019). In plantation areas, resource availability may be limited by extensive disturbance (De Vitis et al. 2020), and the findings of our fieldwork in this study indicated that during the early stages of afforestation, plantation communities are dominated by grass species. However, shrub species were better able to compete with grass species during this stage of forest development (Barnes and Archer 1999; Köchy and Wilson 2000). In this regard, the niche complementarity hypothesis states that diverse species assemblages achieve higher productivity on account of the fact that they generally comprise complementary species (Cipriotti et al. 2014; Mensah et al. 2018). Some possible scenarios of interactions between drivers and feedback could explain the transition from one stage to the next and the potential irreversibility of the shift from grass to shrub dominance (Cardinale et al. 2002; Forrester and Tang 2016). Grass species richness may be lower as a consequence of strong competition from tree species (Biro et al. 2024). Furthermore, competitive exclusion in the context of shrubs is a phenomenon in which one shrub species outcompetes another within the same environment, leading to the eventual dominance of the more successful species (Wojcikiewicz et al. 2024). Shrub species with a stronger competitive advantage (e.g., better resource utilization, growth rate, or tolerance to environmental stress) eliminate other species from plantation areas during the early stage of afforestation (García‐Nieto et al. 2013; Acharya et al. 2019; Geissler et al. 2019). Negative correlations with shrub diversity may occur during the early afforestation of alpine regions.

During afforestation, ecosystem functions and services may benefit from shrub encroachment (Cardinale et al. 2002; García‐Nieto et al. 2013; Maestre et al. 2016). The effects of shrub encroachment on ecosystem structure and function, either positive or negative, are strongly dependent on the functional traits of the encroaching shrubs being displaced (Maestre et al. 2016; Wan et al. 2024). Shrubs are vital components of forest ecosystems that play an important role in maintaining species diversity, promoting nutrient cycling, protecting regenerating seedlings, and fostering multi‐trophic interactions (García‐Nieto et al. 2013; Zhou et al. 2019). They also contribute to soil fertility, water retention, and stabilization, whilst modifying microclimatic conditions and providing conditions conducive to the development of a diverse understory (Eldridge et al. 2011; Zhou et al. 2019). In addition, some shrubs contribute to an enhancement of nutrient pools through nitrogen fixation (Eldridge et al. 2011; Maestre et al. 2016; Wan and Wang 2023). Hence, the growth and distribution of shrub species should be monitored during the early stages of afforestation to optimize plant diversity and ecosystem functions, which ties in with our objective of this study to establish approaches for enhancing plant diversity and restoring ecological functions by regulating shrub biomass during the early stages of afforestation.

An important finding of this study was that shrub biomass has a significant effect on PD, a comparable and evolutionary measure of biodiversity that cannot be evaluated based solely on species count data (Rodrigues and Gaston 2002; Winter et al. 2013). PD is indicative of functional diversity, which in turn is closely associated with ecosystem function (Winter et al. 2013). In alpine regions, afforestation may have a substantial influence on the composition and quality of soil organic matter (Cogliastro et al. 2003; Korkanç 2014). PD has been established to be an effective driver of soil multifunctionality, followed by functional diversity, and is closely associated with certain soil characteristics such as nutrient cycling and microbial communities (Pio et al. 2011; Winter et al. 2013). A higher PD is generally associated with a higher soil fertility and diverse microbial communities, as diverse plant communities release a wider range of organic matter that is conducive to the growth and activity of a more diverse range of microbial species (Flynn et al. 2011; Winter et al. 2013; Fang et al. 2024). Furthermore, PD can enhance soil carbon sequestration and sustain plant diversity and productivity, and it is conceivable that during the early stages of afforestation, the association between PD and ecosystem function may be influenced by shrub biomass (Flynn et al. 2011).

In addition, we demonstrated that the effects of biomass on SD and PD are dependent on different spatial scales (i.e., plot and site) and life forms (i.e., trees or shrubs), thereby providing further evidence regarding the efficacy of afforestation‐based ecological restoration. Our findings revealed that at the site scale, tree biomass had strong positive effects on SD, whereas at the plot scale, shrub biomass had negative effects on SD and PD. The diversity of plants is determined by the availability of resources, which tends to be low during the early stages of afforestation (De Vitis et al. 2020). In alpine regions, however, the establishment of plantations can enhance shrub encroachment (Maestre et al. 2016; Zhou et al. 2019). Moreover, in alpine ecosystems, shrub species have a notable tendency to become established in plantation communities, facilitated by increases in resource availability attributable to planted trees, and tend to be competitively dominant to herb species, thereby potentially leading to a reduction in plant diversity at smaller scales (represented by the plot scale in the present study; Barnes and Archer 1999; Köchy and Wilson 2000; Hartley 2002; Geissler et al. 2019; Zhou et al. 2019). However, during the early stages of afforestation, planting density and the size of individual trees should be effectively managed to enhance the plant diversity of plantation sites (Hartley 2002). Hence, it is conceivable that during the early stages of afforestation, the regulation of tree and shrub biomass drives plant diversity at different spatial scales. In this regard, our findings indicate that in suitable areas within alpine regions, we can regulate tree and shrub biomass to promote the recovery of plant diversity, as indicated by SD and PD.

Although our findings have yielded valuable data with respect to tree species selection and afforestation planning in high‐altitude areas, the present study does have certain limitations. Firstly, we did not consider whether planting density and environmental conditions (such as soil type and climate) would have a substantial influence on biomass diversity relationships. Plant density may be largely dependent on the basal diameter, diameter at breast height, and height of trees, and in our analyses, we did not take into account planting density. Moreover, we focused specifically on the biomass regulation of plant diversity at small spatial scales (i.e., 1 × 1 m^2^ and 10 × 10 m^2^), and it is conceivable that biotic interactions play a more important role than regional or local environmental conditions (Wan et al. 2024). Future studies should accordingly consider changes in biomass diversity relationships at different planting densities and under different environmental conditions.

## 5 Conclusions

We conclude that during early stage afforestation, plant diversity is regulated by the biomass of trees and shrubs. However, the effects of tree biomass on plant diversity (i.e., initially increasing and subsequently declining) differed from those of shrubs (i.e., a negative effect). The effects of biomass on SD and PD were found to be dependent on different spatial scales (i.e., plot and site) and life forms (i.e., tree or shrub), thereby providing further evidence regarding plant diversity in alpine regions likely to be effectively restored by afforestation. Hence, it is possible that tree and shrub biomass can be used to predict plant diversity (i.e., SD and PD) in alpine plantations. Our findings in this study provide new empirical reference data for tree species selection and the planning of planting strategies in alpine regions.

## Author Contributions

**Chun‐Jing Wang:** conceptualization (equal), data curation (equal), formal analysis (equal), funding acquisition (equal), investigation (equal), methodology (equal), project administration (equal), resources (equal), software (equal), supervision (equal), validation (equal), visualization (equal), writing – original draft (equal), writing – review and editing (equal). **Wu‐Xian Yan:** software (equal), validation (equal), visualization (equal). **Shan‐Feng Huang:** resources (equal). **Dong‐Zhou Deng:** funding acquisition (equal), project administration (equal).

## Conflicts of Interest

The authors declare no conflicts of interest.

## Supporting information


Data S1.

## Data Availability

The data that support the findings of this study are available from the Science Data Bank (https://doi.org/10.57760/sciencedb.20996) and Table S3.
