# Supplementary material for: Linking Tree and Shrub Biomass to Plant Diversity During Early‐Stage Alpine Afforestation
Source: Ecol Evol. 2025 Aug 27;15(9):e71842. doi: 10.1002/ece3.71842 (PMC12391032; doi:10.1002/ece3.71842)
Supplement: Supplementary file 1 — Data S1. [file ECE3-15-e71842-s001.docx]

**Supporting information**

Table S1. Biomass equations for trees in our study based on the study of Luo et al. (2020). W: biomass; D: diameter at breast height; H: height.

| Species | Family | Genus | Equation | Units | | |
| --- | --- | --- | --- | --- | --- | --- |
| J*uniperus formosana* Hayata, 1908 | Cupressaceae | Juniperus | W=a+b*(D^2*H) | W/kg | D/cm | H/m |
| *Salix matsudana* Koidz., 1915 | Salicaceae | Salix | W=a*D^b | W/kg | D/cm |  |
| *Ulmus pumila* Linnaeus, 1753 | Ulmaceae | Ulmus | W=a+b*(D^2*H) | W/kg | D/cm | H/m |
| *Picea crassifolia* Komarov, 1923 | Pinaceae | Picea | W=a*(D^2*H)^b | W/kg | D/cm | H/m |
| *Elaeagnus angustifolia* Linnaeus, 1753 | Elaeagnaceae | Elaeagnus | M=a(D^2^H)^b^ | M/kg | D/cm | H/m |
| *Populus davidiana* Dode, 1905 | Salicaceae | Populus | W=a*(D^2*H)^b | W/kg | D/cm | H/m |
| *Crataegus pinnatifida* Bunge, 1835 | Rosaceae | Crataegus | M=a(DH)^b^ | M/kg | D/cm | H/m |
| *Populus simonii* Carrière, 1867 | Salicaceae | Populus | W=a*(D^2*H)^b | W/kg | D/cm | H/m |
| *Populus alba* var. *pyramidalis* Bunge, 1854 | Salicaceae | Populus | W=a*(D^2*H)^b | W/kg | D/cm | H/m |
| *Prunus armeniaca* Linnaeus, 1753 | Rosaceae | Prunus | M=a(D^2^H)^b^ | M/kg | D/cm | H/m |
| *Prunus pseudocerasus* Lindl.,1830 | Rosaceae | Prunus | M=a(D^2^H)^b^ | M/kg | D/cm | H/m |
| *Pinus tabuliformis* Carrière, 1867 | Pinaceae | Pinus | ln(W)=a+b*ln(D^2*H) | W/kg | D/cm | H/m |
| *Ulmus pumila* Linnaeus, 1753 | Ulmaceae | Ulmus | W=a*D^b | W/kg | D/cm |  |

Table S2. Biomass equations for shrubs in our study based on the study of Wang et al. (2021). M: biomass; D: diameter at base height; H: height.

| Species | Family | Genus | Equation | Units | | |
| --- | --- | --- | --- | --- | --- | --- |
| *Syringa reticulata* subsp. amurensis (Rupr.) P. S. Green & M. C. Chang, 1983 | Oleaceae | Syringa | M=aD_10_^b^ | M/kg | D/cm | H/m |
| *Lonicera hispida* Pall. ex Roem. & Schult., 1819 | Caprifoliaceae | Lonicera | M=a(D^2^H)^b^ | M/kg | D/cm | H/m |
| *Spiraea alpina* Pallas, 1784 | Rosaceae | Spiraea | M=a+b×P^2^H | M/g |  | H/m |
| *Lonicera rupicola* var. *syringantha* (Maxim.) Zabel, 1903 | Caprifoliaceae | Lonicera | M=a(D^2^H)^b^ | M/kg | D/cm | H/m |
| *Caragana tibetica* Komarov, 1909 | Fabaceae | Caragana | M=aA_c_^b^ | M/kg | A_c_/m^2^ |  |
| *Caragana korshinskii* Komarov, 1909 | Fabaceae | Caragana | M=aA_c_^b^N^c^ | M/kg | A_c_/m^2^ |  |
| *Myricaria paniculata* P. Y. Zhang, 1984 | Tamaricaceae | Myricaria | M=a+b×CH+c×(CH)^2^ | M/g | C/cm | H/cm |
| *Hippophae rhamnoides* Linn. subsp. sinensis Rousi, 1971 | Elaeagnaceae | Hippophae | M=a(D^2^H)^b^ | M/kg | D/cm | H/m |
| *Salix oritrepha* C. K. Schneid., 1916 | Salicaceae | Salix | M=a(D^2^H)^b^ | M/kg | D/cm | H/m |
| *Berberis vernae* Schneid., 1913 | Berberidaceae | Berberis | M=a(D^2^H)^b^ | M/g | D/cm | H/m |
| *Cotoneaster multiflorus* Bunge, 1830 | Rosaceae | Cotoneaster | M=a(D^2^H)^b^ | M/kg | D/cm | H/m |
| *Salix taoensis* Goerz ex Rehder & Kobuski, 1932 | Salicaceae | Salix | M=a+b×P^2^H | M/g |  | H/m |
| *Daslphora parvifolla* (Fisch.) Juzep, 1941 | Rosaceae | Dasiphora | M=a+b×P^2^H | M/g |  | H/m |
| *Dasiphora glabra* (G. Lodd.) Soják, 1983 | Rosaceae | Dasiphora | M=a+b×P^2^H | M/g |  | H/m |
| *Prunus triloba* Lindley, 1857 | Rosaceae | Prunus | M=a(D^2^H)^b^ | M/kg | D/cm | H/m |
| *Sibiraea angustata* (Rehder) Hand.-Mazz., 1933 | Rosaceae | Sibiraea | M=a+b×P^2^H | M/g |  | H/m |
| *Sorbaria sorbifolia* (L.) A. Braun, 1864 | Rosaceae | Sorbaria | M=a(D^2^H)^b^ | M/kg | D/cm | H/m |
| *Salix sinica* (Hao) C. Wang et C. F. Fang, 1936 | Salicaceae | Salix | M=a(D^2^H)^b^ | M/kg | D/cm | H/m |
| *Syringa oblata* Lindley, 1859 | Oleaceae | Syringa | M=a(D^2^H)^b^ | M/kg | D/cm | H/m |
| *Amorpha fruticosa* Linnaeus, 1753 | Fabaceae | Amorpha | M=a+b×D^2^H | M/kg | D/cm | H/m |

Table S3. Data on diversity indexes including species diversity (SD) (i.e., the Shannon index, Simpson’s index, species richness, and Pielou’s evenness) and phylogenetic diversity (PD) (i.e., Faith’s PD and net relatedness index [NRI]) at the site and plot scales used in our study. All the data on SD, PD, and biomass were log transformed in this table.

| SiteCode | Area | siteShannon | siteSimpson | siteRichness | sitePielou | sitePD | siteNRI | plotShannon | plotSimpson | plotRichness | plotPielou | plotPD | plotNRI |
| --- | --- | --- | --- | --- | --- | --- | --- | --- | --- | --- | --- | --- | --- |
| 1 | A1 | 1.14 | -0.06 | 3.53 | -0.12 | 7.82 | 0.23 | 0.85 | -0.16 | 2.91 | -0.22 | 7.40 | 0.03 |
| 2 | A1 | 1.10 | -0.07 | 3.56 | -0.17 | 8.01 | 0.00 | 0.76 | -0.21 | 2.80 | -0.26 | 7.40 | -0.34 |
| 3 | A1 | 1.10 | -0.07 | 3.53 | -0.16 | 7.96 | 0.00 | 0.77 | -0.18 | 2.69 | -0.22 | 7.32 | -0.21 |
| 4 | A1 | 1.01 | -0.12 | 3.64 | -0.29 | 8.14 | 0.00 | 0.67 | -0.24 | 2.76 | -0.34 | 7.58 | -0.95 |
| 5 | A1 | 0.24 | -0.64 | 3.04 | -0.87 | 7.80 | 0.00 | 0.10 | -0.72 | 2.12 | -0.65 | 7.18 | 0.00 |
| 6 | A1 | 0.80 | -0.16 | 2.89 | -0.27 | 7.43 | -0.63 | 0.26 | -0.43 | 1.82 | -0.33 | 6.91 | -1.72 |
| 7 | A2 | 0.51 | -0.40 | 2.94 | -0.57 | 7.62 | 0.00 | 0.15 | -0.67 | 2.05 | -0.56 | 7.06 | -1.20 |
| 8 | A2 | 0.50 | -0.38 | 2.64 | -0.47 | 7.53 | 0.00 | 0.33 | -0.48 | 2.17 | -0.44 | 7.12 | -2.04 |
| 9 | A2 | 0.84 | -0.18 | 2.94 | -0.24 | 7.64 | 0.00 | 0.41 | -0.37 | 1.92 | -0.24 | 6.99 | -1.99 |
| 10 | A2 | -0.40 | -0.73 | 0.69 | -0.03 | 6.38 | -1.61 | 0.09 | -0.59 | 1.67 | -0.39 | 6.78 | -0.28 |
| 11 | A2 | 0.92 | -0.13 | 3.18 | -0.23 | 7.76 | 0.00 | 0.64 | -0.23 | 2.29 | -0.19 | 7.13 | -0.66 |
| 12 | A2 | 0.51 | -0.39 | 2.77 | -0.51 | 7.41 | -0.79 | 0.22 | -0.61 | 2.01 | -0.47 | 6.97 | -0.61 |
| 13 | A3 | 0.31 | -0.46 | 2.64 | -0.66 | 7.48 | 0.00 | 0.32 | -0.36 | 1.64 | -0.17 | 6.93 | -0.46 |
| 14 | A3 | 0.51 | -0.31 | 2.48 | -0.40 | 7.41 | 0.00 | -0.03 | -0.82 | 1.77 | -0.60 | 7.03 | -0.10 |
| 15 | A3 | 0.72 | -0.29 | 2.89 | -0.34 | 7.68 | 0.00 | 0.45 | -0.32 | 2.03 | -0.25 | 7.14 | 0.05 |
| 16 | A3 | 0.59 | -0.37 | 3.00 | -0.50 | 7.62 | -0.43 | 0.48 | -0.31 | 1.98 | -0.20 | 7.08 | -0.84 |
| 17 | A3 | 0.47 | -0.40 | 2.83 | -0.57 | 7.60 | -1.73 | 0.34 | -0.46 | 1.95 | -0.32 | 7.05 | -0.21 |
| 18 | A3 | -0.14 | -0.73 | 1.39 | -0.46 | 6.74 | 0.00 | -1.08 | -1.60 | 0.55 | -0.78 | 6.36 | 0.00 |
| 19 | A4 | 0.54 | -0.29 | 2.56 | -0.40 | 7.37 | -2.23 | 0.14 | -0.47 | 1.41 | -0.19 | 6.69 | -0.56 |
| 20 | A4 | 0.20 | -0.66 | 2.40 | -0.68 | 7.27 | -1.01 | -0.94 | -1.55 | 0.86 | -0.75 | 6.48 | -0.84 |
| 21 | A4 | 0.52 | -0.36 | 3.00 | -0.58 | 7.65 | 0.00 | -0.42 | -1.16 | 1.42 | -0.72 | 6.74 | -1.24 |
| 22 | A4 | 0.58 | -0.36 | 2.83 | -0.46 | 7.61 | 0.00 | -0.40 | -1.05 | 1.35 | -0.68 | 6.69 | -0.50 |
| 23 | A4 | 0.44 | -0.34 | 2.48 | -0.47 | 7.50 | 0.00 | -0.39 | -1.01 | 1.23 | -0.56 | 6.67 | 0.00 |
| 24 | A4 | -0.01 | -0.75 | 2.30 | -0.85 | 7.36 | 0.00 | -0.62 | -1.26 | 1.10 | -0.65 | 6.59 | -1.07 |
| 25 | A5 | 0.79 | -0.20 | 3.22 | -0.38 | 7.79 | 0.00 | 0.46 | -0.32 | 2.15 | -0.30 | 7.09 | -0.53 |
| 26 | A5 | 0.61 | -0.29 | 3.22 | -0.55 | 7.71 | 0.00 | 0.17 | -0.64 | 2.12 | -0.58 | 7.08 | -0.87 |
| 27 | A5 | 1.01 | -0.11 | 3.53 | -0.25 | 7.92 | -0.37 | 0.63 | -0.24 | 2.50 | -0.28 | 7.19 | -0.21 |
| 28 | A5 | 0.70 | -0.27 | 3.09 | -0.43 | 7.76 | 0.00 | 0.51 | -0.30 | 2.22 | -0.29 | 7.13 | 0.15 |
| 29 | A5 | 0.96 | -0.16 | 3.56 | -0.31 | 7.94 | 0.00 | 0.34 | -0.53 | 2.28 | -0.48 | 7.07 | -0.34 |
| 30 | A5 | 0.78 | -0.27 | 3.33 | -0.42 | 7.74 | 0.20 | 0.44 | -0.39 | 2.31 | -0.40 | 7.18 | -0.63 |
| 31 | A6 | 0.77 | -0.21 | 3.26 | -0.41 | 7.69 | 0.00 | 0.62 | -0.24 | 2.40 | -0.25 | 7.08 | -0.21 |
| 32 | A6 | 1.07 | -0.07 | 3.53 | -0.19 | 7.98 | 0.00 | 0.64 | -0.24 | 2.55 | -0.29 | 7.21 | -0.40 |
| 33 | A6 | 1.00 | -0.12 | 3.53 | -0.26 | 7.92 | 0.00 | 0.60 | -0.30 | 2.45 | -0.29 | 7.24 | -0.62 |
| 34 | A6 | 1.02 | -0.08 | 3.30 | -0.17 | 7.67 | 0.08 | 0.75 | -0.21 | 2.62 | -0.21 | 7.28 | -0.49 |
| 35 | A6 | 1.00 | -0.12 | 3.50 | -0.25 | 7.89 | 0.00 | 0.76 | -0.19 | 2.63 | -0.20 | 7.32 | -0.58 |
| 36 | A6 | 1.04 | -0.08 | 3.37 | -0.17 | 7.81 | -0.33 | 0.77 | -0.18 | 2.66 | -0.21 | 7.38 | -0.94 |
| 37 | A7 | 0.43 | -0.52 | 2.71 | -0.57 | 7.39 | -0.53 | 0.05 | -0.64 | 1.67 | -0.44 | 6.81 | -0.96 |
| 38 | A7 | 0.50 | -0.37 | 3.00 | -0.60 | 7.55 | -0.26 | -0.23 | -1.07 | 1.91 | -0.86 | 6.88 | -0.49 |
| 39 | A7 | 0.72 | -0.22 | 2.56 | -0.22 | 7.34 | -0.39 | -0.16 | -0.75 | 1.33 | -0.36 | 6.65 | -0.90 |
| 40 | A7 | 0.66 | -0.23 | 2.94 | -0.42 | 7.59 | 0.00 | 0.10 | -0.57 | 1.68 | -0.40 | 6.85 | -0.64 |
| 41 | A7 | 0.58 | -0.30 | 2.89 | -0.48 | 7.53 | 0.00 | -0.32 | -1.15 | 1.78 | -0.89 | 6.89 | -1.29 |
| 42 | A7 | 0.06 | -0.79 | 2.40 | -0.81 | 7.12 | 0.16 | -0.73 | -1.43 | 1.45 | -1.05 | 6.67 | -0.33 |
| 43 | A8 | 0.57 | -0.25 | 2.83 | -0.47 | 7.51 | 0.00 | -0.78 | -1.55 | 1.35 | -1.07 | 6.62 | -0.89 |
| 44 | A8 | 0.11 | -0.45 | 2.08 | -0.62 | 7.04 | -0.81 | -1.91 | -2.80 | 0.96 | -1.80 | 6.42 | -0.72 |
| 45 | A8 | 0.79 | -0.17 | 3.04 | -0.32 | 7.51 | -0.35 | 0.39 | -0.41 | 2.07 | -0.33 | 7.05 | -1.27 |
| 46 | A8 | 0.76 | -0.32 | 3.33 | -0.45 | 7.71 | -0.01 | 0.68 | -0.20 | 2.37 | -0.18 | 7.18 | -0.64 |
| 47 | A8 | 0.84 | -0.23 | 3.37 | -0.37 | 7.84 | -0.24 | 0.44 | -0.38 | 2.42 | -0.43 | 7.25 | -0.86 |
| 48 | A8 | 0.92 | -0.14 | 3.30 | -0.27 | 7.68 | -0.03 | 0.49 | -0.36 | 2.32 | -0.34 | 7.11 | -0.28 |
| 49 | A9 | 0.70 | -0.25 | 2.89 | -0.36 | 7.50 | -0.09 | -0.22 | -0.92 | 1.76 | -0.77 | 6.73 | -0.15 |
| 50 | A9 | 0.49 | -0.37 | 3.00 | -0.61 | 7.59 | 0.00 | 0.05 | -0.60 | 1.59 | -0.41 | 6.73 | -0.89 |
| 51 | A9 | 0.80 | -0.22 | 3.04 | -0.31 | 7.59 | 0.00 | 0.40 | -0.44 | 2.18 | -0.37 | 7.00 | -0.06 |
| 52 | A9 | 0.83 | -0.20 | 3.14 | -0.31 | 7.63 | -1.92 | 0.19 | -0.59 | 2.01 | -0.50 | 6.74 | 0.55 |
| 53 | A9 | 0.50 | -0.32 | 2.71 | -0.49 | 7.37 | -0.51 | -0.10 | -0.75 | 1.54 | -0.51 | 6.66 | -0.67 |
| 54 | A9 | 0.73 | -0.18 | 2.71 | -0.26 | 7.28 | 0.07 | 0.19 | -0.55 | 1.96 | -0.48 | 6.81 | 0.06 |
| 55 | A10 | 0.68 | -0.25 | 3.18 | -0.48 | 7.55 | 0.31 | 0.13 | -0.60 | 2.02 | -0.56 | 6.89 | -0.90 |
| 56 | A10 | 0.76 | -0.25 | 3.04 | -0.35 | 7.62 | 0.00 | 0.23 | -0.50 | 1.81 | -0.36 | 6.83 | -0.88 |
| 57 | A10 | 0.42 | -0.39 | 2.64 | -0.55 | 7.41 | 0.00 | 0.00 | -0.62 | 1.59 | -0.46 | 6.59 | 0.05 |
| 58 | A10 | 0.80 | -0.17 | 3.30 | -0.39 | 7.74 | 0.00 | 0.38 | -0.49 | 2.22 | -0.41 | 6.99 | -0.80 |
| 59 | A11 | 0.87 | -0.15 | 3.04 | -0.25 | 7.63 | 0.00 | 0.41 | -0.43 | 2.26 | -0.40 | 7.02 | -0.07 |
| 60 | A11 | 0.39 | -0.54 | 3.18 | -0.76 | 7.68 | 0.00 | 0.45 | -0.43 | 2.46 | -0.45 | 7.33 | -0.11 |
| 61 | A11 | 0.87 | -0.16 | 3.18 | -0.29 | 7.67 | -0.09 | 0.26 | -0.52 | 2.05 | -0.45 | 7.06 | -1.00 |
| 62 | A11 | 0.72 | -0.23 | 3.26 | -0.47 | 7.72 | 0.27 | 0.41 | -0.39 | 2.14 | -0.34 | 7.03 | -0.55 |
| 63 | A11 | 0.72 | -0.21 | 2.94 | -0.36 | 7.54 | 0.03 | 0.42 | -0.35 | 2.07 | -0.31 | 7.08 | -1.06 |
| 64 | A11 | 0.43 | -0.42 | 3.14 | -0.71 | 7.74 | -0.28 | -0.15 | -0.94 | 1.83 | -0.74 | 6.96 | -0.97 |
| 65 | A12 | 0.88 | -0.17 | 3.61 | -0.41 | 8.09 | 0.00 | 0.68 | -0.25 | 2.79 | -0.35 | 7.54 | -1.49 |
| 66 | A12 | 0.81 | -0.17 | 3.47 | -0.43 | 8.08 | 0.00 | 0.50 | -0.38 | 2.50 | -0.42 | 7.38 | -1.55 |
| 67 | A12 | 0.80 | -0.26 | 3.26 | -0.38 | 7.89 | 0.00 | 0.57 | -0.31 | 2.46 | -0.33 | 7.34 | -0.50 |
| 68 | A12 | 0.89 | -0.13 | 3.50 | -0.36 | 8.03 | 0.00 | 0.61 | -0.29 | 2.64 | -0.36 | 7.51 | 0.00 |
| 69 | A12 | 1.08 | -0.11 | 3.83 | -0.26 | 8.26 | 0.00 | 0.87 | -0.15 | 2.94 | -0.21 | 7.69 | 0.00 |
| 70 | A12 | 0.66 | -0.27 | 3.37 | -0.56 | 8.00 | 0.00 | 0.46 | -0.40 | 2.47 | -0.44 | 7.36 | -0.21 |
| 71 | A10 | 0.66 | -0.26 | 3.04 | -0.45 | 7.64 | 0.00 | 0.02 | -0.77 | 1.90 | -0.58 | 6.87 | -0.22 |
| 72 | A10 | 0.52 | -0.35 | 2.71 | -0.48 | 7.37 | 0.00 | 0.20 | -0.50 | 1.84 | -0.40 | 6.72 | 0.30 |
| 73 | A13 | 1.15 | -0.08 | 3.78 | -0.19 | 8.20 | 0.00 | 0.80 | -0.17 | 2.91 | -0.26 | 7.63 | 0.00 |
| 74 | A13 | 0.99 | -0.14 | 3.85 | -0.36 | 8.26 | 0.00 | 0.82 | -0.19 | 2.92 | -0.25 | 7.63 | 0.00 |
| 75 | A13 | 0.91 | -0.15 | 3.58 | -0.36 | 8.12 | 0.00 | 0.75 | -0.21 | 2.72 | -0.25 | 7.54 | 0.00 |
| 76 | A13 | 0.86 | -0.15 | 3.26 | -0.32 | 7.57 | 0.00 | 0.45 | -0.36 | 2.22 | -0.34 | 7.00 | -0.27 |
| 77 | A13 | 1.09 | -0.07 | 3.71 | -0.23 | 8.00 | 0.00 | 0.69 | -0.21 | 2.74 | -0.31 | 7.48 | -0.22 |
| 78 | A13 | 0.93 | -0.13 | 3.53 | -0.33 | 7.88 | 0.00 | 0.71 | -0.20 | 2.77 | -0.30 | 7.44 | -0.10 |
